# Pyruvate dehydrogenase kinase 1 and 2 deficiency reduces high-fat diet-induced hypertrophic obesity and inhibits the differentiation of preadipocytes into mature adipocytes

**DOI:** 10.1038/s12276-021-00672-1

**Published:** 2021-09-22

**Authors:** Hyeon-Ji Kang, Byong-Keol Min, Won-Il Choi, Jae-Han Jeon, Dong Wook Kim, Sungmi Park, Yun-Kyung Lee, Hwa-jin Kim, Ju-Eun Byeon, Younghoon Go, Hye Jin Ham, Yong Hyun Jeon, Mi-Jin Kim, Jung Yi Lee, Adam R. Wende, Sung Hee Choi, Robert A. Harris, In-Kyu Lee

**Affiliations:** 1 Research Institute of Aging and Metabolism, Kyungpook National University, Daegu, Republic of Korea; 2 Arbormed Co., Ltd., Seoul, Republic of Korea; 3 Graduate School of Medical Science and Engineering, Korea Advanced Institute of Science and Technology, Daejeon, Republic of Korea; 4 Leading-edge Research Center for Drug Discovery and Development for Diabetes and Metabolic Disease, Kyungpook National University Hospital, Daegu, Republic of Korea; 5 Department of Internal Medicine, School of Medicine, Kyungpook National University, Kyungpook National University Chilgok Hospital, Daegu, Republic of Korea; 6 Department of Biochemistry, College of Veterinary Medicine, Seoul National University, Seoul, Republic of Korea; 7 Department of Internal Medicine, Seoul National University Bundang Hospital, Seongnam, Republic of Korea; 8 BK21 Plus KNU Biomedical Convergence Programs, Department of Biomedical Science, Kyungpook National University, Daegu, Republic of Korea; 9 Department of Biomedical Science, Graduate School, Kyungpook National University, Daegu, Republic of Korea; 10 Korean Medicine Application Center, Korea Institute of Oriental Medicine, Daegu, Republic of Korea; 11 Laboratory Animal Center, Daegu-Gyeongbuk Medical Innovation Foundation, Daegu, Republic of Korea; 12 Division of Molecular and Cellular Pathology, Department of Pathology, University of Alabama at Birmingham, Birmingham, AL USA; 13 Department of Biochemistry and Molecular Biology, The University of Kansas Medical Center, Kansas City, KS USA; 14 Department of Internal Medicine, School of Medicine, Kyungpook National University, Kyungpook National University Hospital, Daegu, Republic of Korea

**Keywords:** Mechanisms of disease, Obesity

## Abstract

Obesity is now recognized as a disease. This study revealed a novel role for pyruvate dehydrogenase kinase (PDK) in diet-induced hypertrophic obesity. Mice with global or adipose tissue-specific PDK2 deficiency were protected against diet-induced obesity. The weight of adipose tissues and the size of adipocytes were reduced. Adipocyte-specific PDK2 deficiency slightly increased insulin sensitivity in HFD-fed mice. In studies with 3T3-L1 preadipocytes, PDK2 and PDK1 expression was strongly increased during adipogenesis. Evidence was found for epigenetic induction of both PDK1 and PDK2. Gain- and loss-of-function studies with 3T3-L1 cells revealed a critical role for PDK1/2 in adipocyte differentiation and lipid accumulation. PDK1/2 induction during differentiation was also accompanied by increased expression of hypoxia-inducible factor-1α (HIF1α) and enhanced lactate production, both of which were absent in the context of PDK1/2 deficiency. Exogenous lactate supplementation increased the stability of HIF1α and promoted adipogenesis. PDK1/2 overexpression-mediated adipogenesis was abolished by HIF1α inhibition, suggesting a role for the PDK-lactate-HIF1α axis during adipogenesis. In human adipose tissue, the expression of PDK1/2 was positively correlated with that of the adipogenic marker PPARγ and inversely correlated with obesity. Similarly, PDK1/2 expression in mouse adipose tissue was decreased by chronic high-fat diet feeding. We conclude that PDK1 and 2 are novel regulators of adipogenesis that play critical roles in obesity.

## Obesity: Effects of different forms of key enzyme

The discovery that two forms of a key enzyme appear to play a critical role in fat production triggered by overeating might lead to new approaches to prevent and treat obesity. Hyeon-Ji Kang at Kyungpook National University, Daegu, South Korea, and colleagues in South Korea and the USA examined the role of the enzymes pyruvate dehydrogenase kinase types 1 and 2 (PDK1/2). PDK enzymes regulate the activity of a multi-enzyme complex that catalyzes a key step in the use of glucose to provide energy stores for cells. Mice deficient in PDK2 were protected from diet-induced obesity, and PDK 1 and 2 activity was increased during the generation of fat cells. Studies using mice and human fat tissue confirmed that the enzymes regulate the development and growth of fat cells. Drugs inhibiting PDK enzymes might combat obesity.

## Introduction

Obesity is an epidemic in the United States. After decades of study, thousands of publications, and a number of clinical trials, obesity remains—as it has always been—easy to diagnose but impossible to treat for want of an effective pharmacological intervention. A better understanding of the molecular mechanisms controlling the function of adipocytes may provide the insight needed to avoid the negative metabolic consequences and health risks associated with obesity.

The capacity of adipocytes to store triacylglycerol and release free fatty acids, the primary function of adipose tissue, is required for survival during starvation, provides the energy needed for physically demanding work and meets the challenges imposed by the stresses of illnesses and infections. By virtue of their ability to expand, adipocytes also provide a temporary storage depot for energy after overconsumption of food. Pyruvate dehydrogenase kinase 2 (PDK2), one of the four mitochondrial kinases responsible for phosphorylation-mediated regulation of the pyruvate dehydrogenase complex, is required for maintenance of euglycemia during starvation but contributes to hyperglycemia in diabetes^1^.

Inhibition or genetic deletion of PDK2 lowers blood glucose and improves insulin sensitivity in diabetic models^2,3^, increases apoptosis and decreases proliferation and tumor growth^4^, suppresses hypoxia-inducible factor-1α (HIF1α) signaling and angiogenesis in cancer^5^, suppresses macrophage polarization toward the M1 phenotype^6^, and is required for induction of apoptosis by p53^4,7^.

This study follows from previous findings that PDK2 deficiency reduces body weight gain, prevents hepatic steatosis, and increases hepatic fatty acid oxidation, ketogenesis, and energy expenditure in mice fed a high-fat diet^2^. Decreased accumulation of body fat was apparent from the lower body weights of PDK2 knockout mice. In the present study, this finding was confirmed and extended by the demonstration that adipose-specific PDK2 deficiency decreased body fat accumulation and the size of adipocytes and improved insulin sensitivity in high-fat diet (HFD)-fed mice. Upregulation of PDK2 along with PDK1 was also found to occur and to be required for full differentiation of 3T3-L1 adipocytes and primary stromal vascular cells into adipocytes. It is proposed that the metabolic effects of PDK2 deficiency reduce obesity by limiting full differentiation of preadipocytes into adipocytes, which in turn limits adipocyte hypertrophy.

## Materials and methods

### Animal study

*Pdk2*^*lox/+*^ mice were generated on a C57BL/6 background by Cyagen Bioscience (Guangzhou, China). The targeting construct included LoxP sites flanking exon 2 of *Pdk2* as well as a neomycin resistance cassette flanked by FRT sites. *Pdk2*^*lox/+*^ mice were intercrossed to generate *Pdk2*^*lox/lox*^ mice. To generate adipocyte-specific PDK2 KO mice, *Pdk2*^*lox/lox*^ mice were crossed with adiponectin-Cre mice. *Pdk2*^*-/-*^ or *Pdk4*^*-/*-^ mice, as well as *Pdk2*^*lox/lox*^ and *Pdk2*^*ad-/-*^ mice, were maintained on a 12-h light/dark cycle at 22 ± 2 °C. Four-week-old mice were fed either a control diet (low-fat diet; 10 kcal% fat; D12450B, Research Diets, Inc., New Brunswick, NJ, USA) or a high-fat diet (HFD; 60 kcal% fat and 6 kcal% sucrose; D12492, Research Diets). The whole-body lean and fat masses of the mice were measured using a MiniSpec LF 50 body composition analyzer (Bruker Optics, Billerica, MA, USA). All animal experiments were approved by the Institutional Animal Care and Use Committee of Kyungpook National University (2015-0063) and the Deagu-Gyeongbuk Medical Innovation Foundation (DGMIF 19020704-00).

### Glucose & insulin tolerance tests (GTT & ITT)

The GTT was performed by injecting mice with D-glucose (1.5 g/kg, i.p.) after 16 h of fasting. Likewise, for the ITT, insulin (0.75 U/kg) was injected i.p. into mice after a 6 h period of fasting. Blood glucose levels were measured with a standard glucometer (Accu-Check Active, Roche Diagnostics GmbH, Mannheim, Germany) using blood collected from cut tail tips at the indicated time points.

### Histological and immunofluorescence analyses

Adipose tissues were fixed with 4% formaldehyde in PBS and embedded in paraffin. Sectioned slides were stained with H&E. The adipocyte area was analyzed using ImageJ software (NIH, Bethesda, MD, USA). For immunofluorescence staining of perilipin, sections were permeabilized with 0.1% Triton X-100 for 15 min at room temperature and incubated with an antibody specific for perilipin A (ab3526, Abcam, Cambridge, MA, USA) in zymogen Ab diluent solution overnight at 4 °C prior to incubation with an Alexa Fluor-568-conjugated anti-rabbit secondary antibody (A11011, Thermo Fisher Scientific, Waltham, MA, USA). Samples were visualized with a confocal microscope (FluoView™ FV1000; Olympus, Tokyo, Japan).

### Cell culture and differentiation

3T3-L1 preadipocytes were obtained from ATCC (Manassas, VA, USA) and maintained in high glucose-DMEM (HyClone, Logan, UT, USA) supplemented with 10% bovine serum (BS, Gibco 16170, Thermo Fisher Scientific) in a humidified atmosphere of 5% CO_2_ at 37 °C. For differentiation, confluent 3T3-L1 preadipocytes were treated with 1 µg/ml insulin (Sigma-Aldrich, St. Louis, MO, USA), 1 µM dexamethasone (Sigma-Aldrich), 0.5 mM IBMX (Sigma-Aldrich), and 10% fetal bovine serum (FBS, Gibco 16000, Thermo Fisher Scientific). After 2 days, the medium was replaced with medium containing 10% FBS and 1 µg/ml insulin and was thereafter replaced every 2 days with medium containing 10% FBS. After 6 days, differentiated 3T3-L1 cells were stained with Oil Red O (Sigma-Aldrich). Oil Red O staining was performed as described previously^8^. Sodium dichloroacetate (DCA, 347795), sodium l-lactate (L7022), and chetomin (C9623) were purchased from Sigma-Aldrich. GSK2837808A (# HY-10031) was purchased from MedChemExpress (Monmouth Junction, NJ, USA).

### Western blot analysis

Western blot analysis was performed as described previously^8^. Proteins were separated by SDS-PAGE, transferred to a PVDF membrane (Millipore, Billerica, MA, USA), and incubated with specific primary antibodies [anti-SREBP-1c (577036, BD Biosciences, Franklin Lakes, NJ, USA), anti-FAS (3180, Cell Signaling, Danvers, MA, USA), anti-C/EBPα (2295, Cell Signaling), anti-PPARγ (2435, Cell Signaling), anti-PDK1 (KAP-PK112, Enzo Life Science, Farmingdale, NY, USA), anti-PDK2 (sc-100534, Santa Cruz, Dallas, TX, USA), anti-PDK3 (sc-365378, Santa Cruz) and anti-PDK4 (ab214938, Abcam), anti-p-PDHE1α Ser232 (AP1063, Calbiochem, San Diego, CA, USA), anti-p-PDHE1α Ser293 (AP1062, Calbiochem), anti-HIF1α (NB100-123, Novus Biologicals, Centennial, CO, USA), anti-HSP90 (4874, Cell Signaling), and anti-β-tubulin (G098, Applied Biological Materials, Richmond, BC, Canada)].

### Real-time PCR

Total RNA was extracted using QIAzol reagent (Qiagen, Germantown, MD, USA) as described by the manufacturer. cDNA was synthesized using a RevertAid^TM^ First Strand cDNA Synthesis Kit (Thermo Fisher Scientific) according to the manufacturer’s instructions. Real-time PCR was performed using SYBR Green (SYBR Green Master Mix, Applied Biosystems, Foster City, CA, USA) in a ViiA 7 Real-Time PCR System (Applied Biosystems). The sequences of the primers used for amplification of mouse and human DNA are shown in Supplementary Table 1.

### Chromatin immunoprecipitation (ChIP) assay

3T3-L1 cells were crosslinked with formaldehyde for 20 min. The reaction was stopped by the addition of 0.25 M glycine for 10 min. Cells were lysed in lysis buffer (1% SDS, 10 mM EDTA, 50 mM Tris-HCl (pH 8)). The lysates were sonicated to generate DNA fragments of 500 to 1000 bp and diluted with ChIP buffer (1% SDS, 1% Triton X-100, 16.7 mM Tris-HCl (pH 8.1), 167 mM NaCl, 1.2 mM EDTA). The samples were incubated with antibodies (anti-H3ac (06–599, Millipore), anti-H4ac (06–598, Millipore), anti-H3K4me3 (07–473, Millipore), anti-PCAF (ab12188, Abcam), anti-p300 (ab14984, Abcam), anti-H3K27ac (ab177178, Abcam) and control mouse IgG (I5381, Sigma-Aldrich)) overnight at 4 °C on a rotating wheel. To collect DNA–protein–antibody complexes, protein A/G-agarose beads were added to the mixtures, which were then incubated at 4 °C for 2 h with rotation and pelleted by brief centrifugation. The samples were washed and dissolved in 500 μl of elution buffer, and crosslinking was reversed by treatment with 20 µl of 5 M NaCl at 65 °C for 2 h. After EDTA and proteinase K treatment, DNA was extracted from the supernatants with phenol/chloroform and precipitated with ethanol. Immunoprecipitated DNA was analyzed by quantitative real-time PCR using primers specific for the mouse PDK1 promoter (forward, 5′-ACAGGCTCATTGCCAACGAT-3′ and reverse, 5′-CCGCCCTCCCAGTCTCA-3′) and the mouse PDK2 promoter (forward, 5′-GGGCACATACCAAGTTTTACCAA-3′ and reverse, 5′-CCCTGCCTCTTCCCTGAGA-3′).

### Retroviral plasmids and retroviral transduction

The full-length cDNAs of PDK1 and PDK2 were amplified by PCR and inserted into the XhoI and NotI sites in the Vxy-puro retroviral vector. The constructs (Vxy-puro, Vxy-*Pdk1*-puro, and Vxy-*Pdk2*-puro) were transfected into Phoenix ecotropic packaging cells using Lipofectamine^TM^ 2000 (Invitrogen, Carlsbad, CA, USA). Viral supernatants were collected after 48 h, clarified by filtration through 0.45-µm pore size syringe filters (Sartorius Stedim Biotech, Bohemia, NY, USA), and used for transduction of 3T3-L1 preadipocytes in the presence of 5 μg/ml polybrene (Sigma-Aldrich) for 24 h. Transduced 3T3-L1 cells were selected with 3 μg/ml puromycin (Sigma-Aldrich) for 7 days.

### Establishment of cells with stable knockdown of *Pdk1* and *Pdk2*

Predesigned short hairpin RNA (shRNA) plasmids targeting *Pdk1* or *Pdk2* were purchased from Qiagen. To generate stable *Pdk1/2* knockdown cells, shRNA-*Pdk1*, shRNA-*Pdk2*, and control vectors were transiently transfected into 3T3-L1 preadipocytes using Attractene Transfection Reagent (Qiagen). Stably transfected cells were selected with puromycin and hygromycin for 7 days.

### Measuring l-Lactate release in cell culture medium

3T3-L1 cell culture medium was collected every 2 days after the differentiation medium was changed. Lactate production was assessed using an EnzyChrom^TM^ l-Lactate Assay Kit (BioAssay Systems, Hayward, CA, USA).

### Human samples

For human studies, visceral adipose tissue was obtained from 34 healthy subjects who underwent nephrectomy as donors for kidney transplantation between approximately 2015 and 2018 (Seoul National University Bundang Hospital, IRB No. B-1801-445-301). Visceral adipose tissues were collected, and correlations between PDK expression, adipogenic genes, and clinical information such as BMI were examined. The baseline characteristics are summarized in Supplementary Table 2.

### Statistical analysis

Data were plotted using GraphPad Prism 8.3 software (GraphPad Inc., San Diego, CA, USA), and statistical analysis was performed using IBM SPSS Statistics (version 21, IBM Corp., Armonk, NY, USA). Statistically significant differences in normally distributed data were identified by 2-tailed Student’s *t* test. Statistical analysis of group comparisons was performed by one-way or two-way ANOVA followed by the LSD post hoc test. *p*-values <0.05 were considered statistically significant.

## Results

### Global *Pdk2* knockout mice exhibit decreased adiposity

**Fig. 1:**
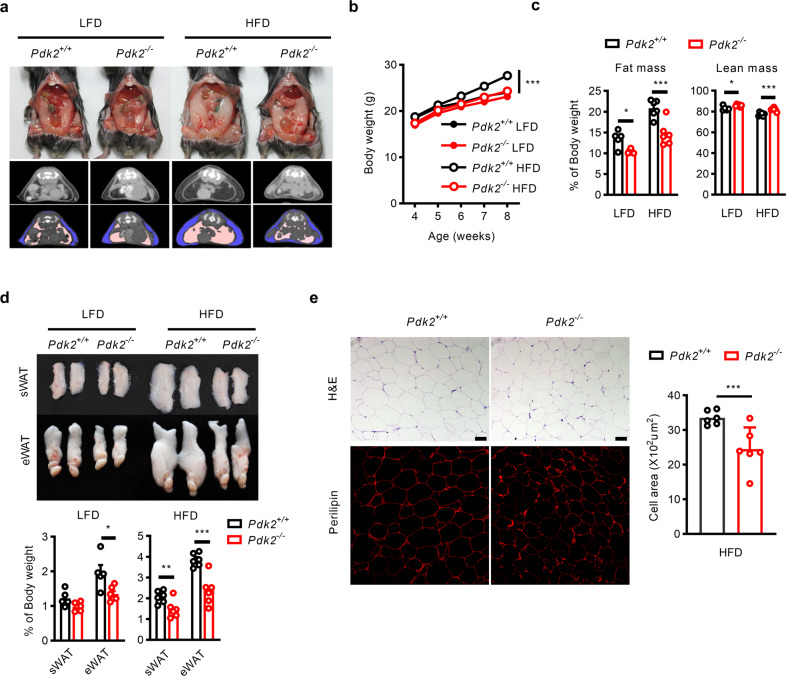
*Pdk2* deficiency inhibits high-fat diet-induced fat accumulation. **a** Representative images and computed tomography (CT) scans revealed fat accumulation in the epididymal adipose tissue of WT and *Pdk2*^*-/-*^ mice fed a LFD or a HFD for 4 weeks. **b** Body weight was monitored in WT and *Pdk2*^*-/-*^ mice fed a LFD or a HFD (*n* = 5–6 per group). **c** Body composition (% of body weight) was analyzed in WT and *Pdk2*^*-/-*^ mice fed a LFD (*n* = 5 per group) or a HFD (*n* = 6 per group) for 3 weeks. **d** Representative morphology and weight (% of body weight) of subcutaneous and epididymal adipose tissue were evaluated in WT and *Pdk2*^*-/-*^ mice fed a LFD or a HFD (*n* = 5–6 per group). **e** H&E and perilipin staining of epididymal adipose tissue from WT and *Pdk2*^*-/-*^ mice fed a HFD for 4 weeks (*n* = 6 per group). The adipocyte area was measured with ImageJ. The data are presented as the mean ± SEM values. *P < 0.05, **P < 0.01, ***P < 0.001 by 1-way ANOVA followed by the LSD test.

*Pdk2*^-/-^ and *Pdk4*^-/-^ mice were used to test the effect of *Pdk2* and *Pdk4* on fat mass. Four-week-old male wild-type (WT), *Pdk2* ^-/-^, and *Pdk4*^-/-^ mice were fed a high-fat diet (HFD) for 6 weeks. Body weight and fat mass (both subcutaneous and epididymal fat) gain were significantly attenuated in *Pdk2*^-/-^ mice but not in *Pdk4*^-/-^ mice compared with WT mice (Supplementary Fig. 1a–c). To systematically assess weight gain in *Pdk2*^-/-^ mice, we fed them either a low-fat diet (LFD) or HFD for 4 weeks. Computed tomography (CT) scans showed less lipid accumulation in visceral (apricot-colored) and subcutaneous (blue-colored) depots in *Pdk2*^-/-^ mice than in WT mice under both LFD and HFD conditions (Fig. 1a). Four weeks after shifting to HFD feeding, *Pdk2*^-/-^ mice exhibited significantly less weight gain than WT mice. Importantly, *Pdk2*^-/-^ mice fed a LFD had body weights comparable to those of their WT counterparts throughout the experiment (Fig. 1b). Body composition analysis revealed that *Pdk2*^*-/-*^ mice showed a slightly decreased fat mass with LFD feeding (13.5 ± 0.9%, *n* = 5 to 10.4 ± 0.2%, *n* = 6), but the reduction was more dramatic when the mice were fed an HFD (20.8 ± 0.9%, *n* = 6 to 14.7 ± 1.2%, *n* = 6; Fig. 1c). No significant changes in the masses of other major metabolic organs, such as brown adipose tissue, the heart, and the kidney, were found between WT and *Pdk2*^-/-^ mice fed either a LFD or a HFD, implying that fat mass is the dominant factor affecting body weight (Supplementary Fig. 1d). The epididymal and subcutaneous fat masses were both smaller in *Pdk2*^*-/-*^ mice than in WT mice fed the HFD (Fig. 1d). Histological examination of epididymal fat tissue by hematoxylin and eosin (H&E) staining indicated that the decrease in fat mass was due to a decrease in adipocyte size, which was further confirmed by staining with the lipid droplet-specific marker perilipin, showing a 26% reduction in adipocyte size (from 33.5 ± 1.1 to 24.8 ± 1.9) under HFD conditions (Fig. 1e); however, the reduction in adipocyte size was less notable under LFD conditions (Supplementary Fig. 1e).

### *Pdk*2 deficiency in adipocytes leads to decreased fat mass in an organ-intrinsic manner

To directly investigate the role of *Pdk*2 in adipose tissues, we used a fat tissue-specific animal model established using the Cre-LoxP recombination system involving Adipoq-Cre and *Pdk2*^*lox/lox*^ mice. As previous studies verified the adipocyte-specific expression pattern of Adipoq-Cre, we established Adipoq-Cre/*Pdk2*^*lox/lox*^ (*Pdk2*^*ad-/-*^) mice, which harbored deletion of exon 2 of the *Pdk*2 gene in all adipocytes (Supplementary Fig. 2a). Western blot analysis demonstrated the efficiency and specificity of *Pdk2* deletion in BAT and WAT but not in nonadipose tissues, such as the liver (Supplementary Fig. 2b).

**Fig. 2:**
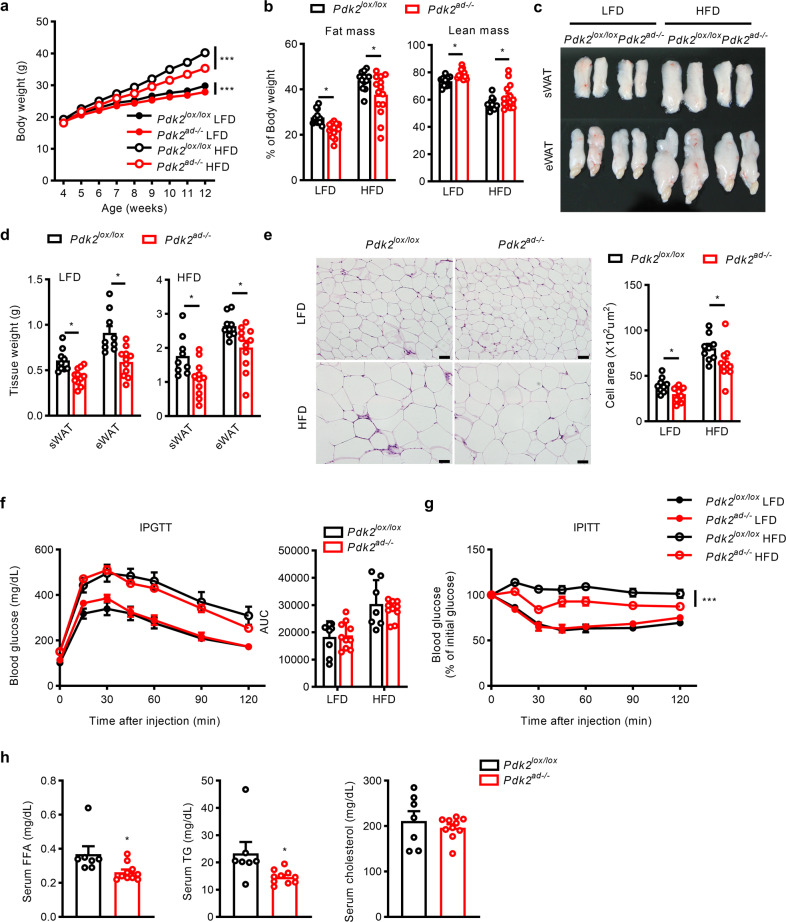
*Pdk2* deficiency in adipose tissue results in decreased fat mass and improved insulin sensitivity. **a** Body weight was measured in *Pdk2*^*lox/lox*^ and *Pdk2*^*ad-/-*^ mice fed a LFD or a HFD (*n* = 12–16 per group). **b** Body composition (% of body weight) was analyzed in *Pdk2*^*lox/lox*^ and *Pdk2*^*ad-/-*^mice fed a LFD or a HFD for 7 weeks (*n* = 12–14 per group). **c** Representative morphology and **d** weight of subcutaneous and epididymal adipose tissue (% of body weight) from *Pdk2*^*lox/lox*^ and *Pdk2*^*ad-/-*^ mice fed a LFD or a HFD for 8 weeks (*n* = 9–11 per group). **e** Representative images of H&E-stained sections of eWAT from *Pdk2*^*lox/lox*^ and *Pdk2*^*ad-/-*^ mice fed a LFD or a HFD for 8 weeks. The adipocyte area was measured with ImageJ. **f** A glucose tolerance test (1.5 g/kg, i.p.) after 16 h of fasting and **g** an insulin tolerance test (0.75 U/kg, i.p.) after 6 h of fasting were performed in *Pdk2*^*lox/lox*^ and *Pdk2*^*ad-/-*^ mice fed a LFD or a HFD (*n* = 7–10 per group). **h** Serum free fatty acid (FFA), triglyceride (TG) and cholesterol levels were measured in *Pdk2*^*lox/lox*^ and *Pdk2*^*ad-/-*^ mice fed a HFD (*n* = 7–10 per group). The data are presented as the mean ± SEM values. *P < 0.05, ***P < 0.001 by 1-way ANOVA followed by the LSD test (**a**–**g**) or by 2-tailed Student’s *t* test (**h**).

When fed a HFD, *Pdk2*^*ad-/-*^ mice appeared much leaner than WT mice, and after 4 weeks on a HFD, *Pdk2*^*ad-/-*^ mice consistently weighed less than WT mice (Fig. 2a). In accordance with the observation in germline *Pdk2*^*-/-*^ mice, the fat mass in *Pdk2*^*ad-/-*^ mice was lower under either LFD or HFD conditions (Fig. 2b). The necropsy data indicated that the size (Fig. 2c) and weight (Fig. 2d) of both subcutaneous and epididymal fat were decreased in *Pdk2*^*ad-/-*^ mice under both HFD and LFD conditions. Histological examination of epididymal fat tissue by H&E staining showed that adipocytes were smaller in *Pdk2*^*ad-/-*^mice fed either a LFD or a HFD (Fig. 2e).

In contrast to HFD-fed global PDK2 knockout mice, which showed dramatic improvements in glucose tolerance and insulin sensitivity^2^, *Pdk2*^*ad-/-*^ mice showed only a slight improvement in insulin sensitivity and no improvement in glucose tolerance (Fig. 2f, g). Serum triglycerides and free fatty acids were also reduced in these mice (Fig. 2h).

To test whether energy expenditure is altered in *Pdk2*^*ad-/-*^ mice, we examined the respiratory exchange ratio, energy expenditure, and physical activity. *Pdk2*^*ad-/-*^ mice showed no significant differences from their counterparts in food intake (Supplementary Fig. 2c) or in VO_2_, VCO_2_, and the corresponding respiratory exchange ratio and energy expenditure (Supplementary Fig. 3), suggesting that the lower fat mass in *Pdk2*^*ad-/-*^ mice is not a consequence of an increased metabolic rate.

### Expression of PDK1 and PDK2 is increased during adipogenesis

Obesity is closely related to excessive accumulation of WAT resulting from both the hypertrophy of preexisting adipocytes and the differentiation of adipocyte precursors into mature adipocytes^9,10^. As both *Pdk2*^*-/-*^ mice and *Pdk2*^*ad-/-*^ mice showed a reduction in adiposity, we reasoned that PDK2 may affect adipocyte differentiation in a cell-intrinsic manner.

**Fig. 3:**
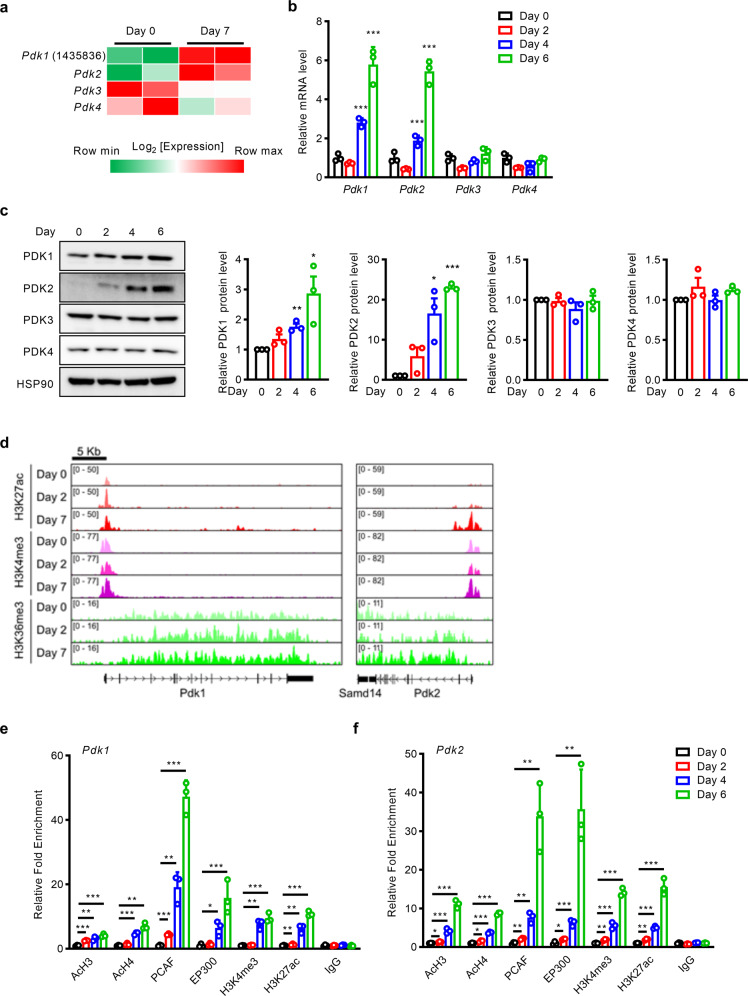
The expression levels of PDK1 and PDK2 are increased during adipogenesis. **a***Pdk* isoform expression levels during 3T3-L1 cell differentiation were analyzed in microarray data. Raw expression microarray data were obtained from GSE20752^11^. The **b** mRNA (*n* = 3) and **c** protein expression levels of PDK isoforms during the differentiation of 3T3-L1 cells were measured by real-time PCR and western blotting, respectively. **d** ChIP‐Seq profiles of H3K27ac, H3K4me3, and H3K36me3 on gene loci encoding *Pdk1* and *Pdk2* during the differentiation of 3T3-L1 cells. Raw expression microarray data were obtained from GSE20752. **e**, **f** AcH3, AcH4, PCAF, EP300, H3K4me3, and H3K27ac recruitment to **e** *Pdk1* and **f** *Pdk2* loci during 3T3-L1 cell differentiation was analyzed by ChIP-qPCR. IgG was used as the negative control (*n* = 3). The data are presented as the mean ± SD values. *P < 0.05, **P < 0.01, ***P < 0.001 by 2-tailed Student’s *t* test.

In agreement with this hypothesis, microarray analysis of differentiated 3T3-L1 cells^11^ showed that *Pdk2* mRNA was highly expressed during 3T3-L1 cell differentiation, especially 7 days after the initiation of differentiation (Fig. 3a). *Pdk*1 also showed an expression pattern similar to that of *Pdk*2 (Fig. 3a).

To further examine the involvement of PDKs in adipogenesis, 3T3-L1 cells were differentiated, and the mRNA and protein expression levels of the *Pdk* isoforms were measured every 2 days by qPCR and western blotting, respectively. In accordance with the microarray data, the protein and mRNA expression levels of *Pdk*1 and *Pdk*2 increased dramatically during differentiation, while those of *Pdk*3 and *Pdk*4 were unaltered (Fig. 3b, c).

To confirm the physiological importance of these findings in 3T3-L1 preadipocytes, we cultured and primary stromal vascular cells and differentiated them into adipocytes. qPCR analysis showed that the expression levels of *Pdk1* and *Pdk2* were increased after differentiation (Supplementary Fig. 4). As observed in 3T3-L1 cells, *Pdk3* and *Pdk4* were not induced during differentiation. These findings show that *Pdk1* and *Pdk2* are upregulated during adipocyte differentiation, suggesting that *Pdk1* and *Pdk2* may play important roles during adipogenesis.

Adipocyte differentiation is an essential process for adipose tissue development, involving complex molecular networks and epigenetic modifications^12,13^. A series of experiments was performed to investigate whether the transcriptional activities of *Pdk1* and *Pdk2* are epigenetically regulated. Published chromatin immunoprecipitation sequencing (ChIP-seq) results^11^ and our own ChIP assay results demonstrated that H3K27 acetylation, which is associated with ‘open’ chromatin (euchromatin) and active *cis*-regulatory regions, was markedly enriched in the region surrounding the transcriptional start site (TSS) in the *Pdk1* and *Pdk2* loci (Fig. 3d). H3K4 trimethylation, which is associated with transcriptional initiation, was slightly increased in differentiated 3T3-L1 cells after 7 days (Fig. 3d). Furthermore, H3K36 trimethylation, which is associated with transcriptional elongation, was distributed across active gene bodies and increased markedly in both *Pdk1* and *Pdk2* as they were upregulated (Fig. 3d). This result suggests that *Pdk1* and *Pdk2* are upregulated via epigenetic modification during adipocyte differentiation.

Histone acetyltransferases (HATs) are recruited to H3K27 for histone acetylation^14–17^. Because recruitment of HATs results in transcriptional activation via H3K27 acetylation, we tested whether HATs are recruited to the *Pdk1* and *Pdk2* promoter regions. The ChIP assay showed that HATs (PCAF and EP300) were markedly recruited to the *Pdk1* and *Pdk2* promoter regions during adipogenesis and that acetylation of both H3 and H4 was dramatically increased in mature 3T3-L1 adipocytes (Fig. 3e, f). The ChIP assay also showed that both H3K4 trimethylation and H3K27 acetylation were distinctly increased, similar to our findings from ChIP-seq (Fig. 3e, f). These results suggest that *Pdk1* and *Pdk2* are highly upregulated during adipocyte differentiation via epigenetic modification.

### PDK1 and PDK2 are critically involved in adipocyte differentiation in 3T3-L1 cells

**Fig. 4:**
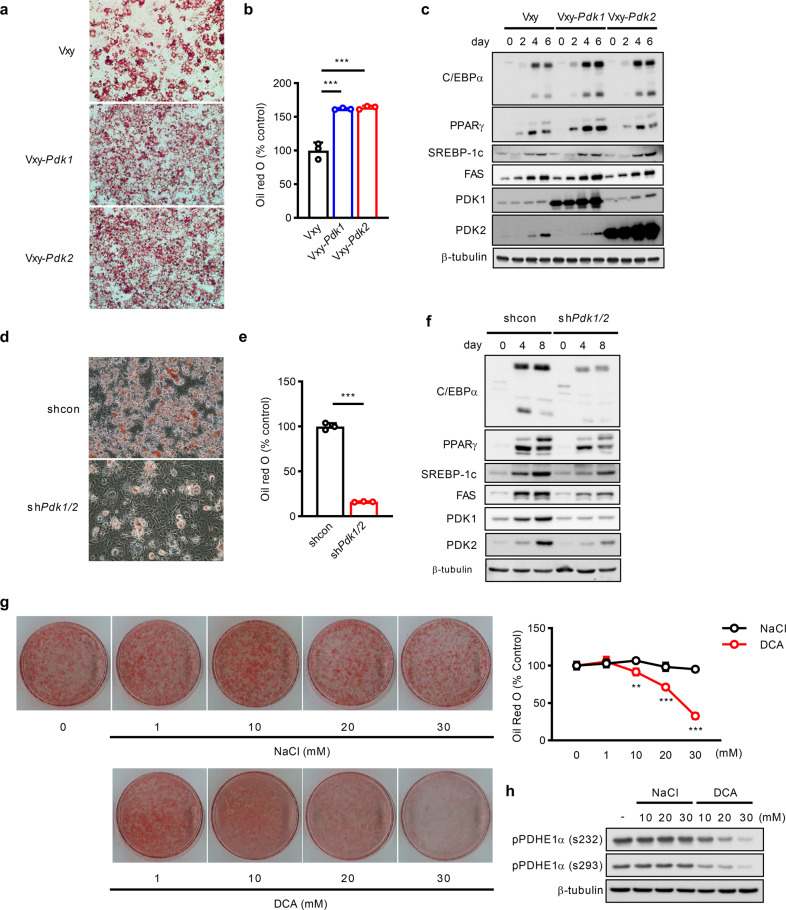
PDKs are required for adipocyte differentiation in 3T3-L1 cells. **a**–**c** 3T3-L1 cells were retrovirally transduced with empty vector (Vxy-puro) or a retroviral vector encoding *Pdk1* or *Pdk2*. **a**, **b** Lipid accumulation in 3T3*-*L1 adipocytes was measured by Oil Red O staining on day 6 after induction of differentiation (*n* = 3). **c** The protein expression levels of adipocyte-specific genes were measured in 3T3-L1 cells stably expressing Vxy-puro, Vxy-*Pdk1*, or Vxy-*Pdk2*. **d**, **e** Lipid accumulation in differentiated control (shcon) and *Pdk1/2*-silenced 3T3-L1 cells (sh*Pdk1/2*) was measured with Oil Red O staining (*n* = 3). **f** The protein expression levels of adipocyte-specific genes were measured in control and *Pdk1/2*-silenced 3T3-L1 cells. **g** Two-day postconfluent 3T3-L1 preadipocytes were cultured in differentiation medium with or without dichloroacetate (DCA), a pan-PDK inhibitor, at the indicated concentration for 6 days. On day 6, intracellular lipid accumulation was evaluated by Oil Red O staining (*n* = 3). **h** The phosphorylation level of PDHE1a was measured in differentiated 3T3-L1 cells. The data are presented as the mean ± SD values. **P < 0.01, ***P < 0.001 by 2-tailed Student’s *t* test.

Next, to test whether PDK1 and PDK2 regulate adipocyte differentiation, 3T3-L1 cells stably expressing *Pdk1* (Vxy-*Pdk1*), *Pdk2* (Vxy-*Pdk2*), or a control vector (Vxy-puro) via a retroviral overexpression system were differentiated into adipocytes. Oil Red O staining showed that differentiated 3T3-L1 cells stably expressing either *Pdk1* or *Pdk2* exhibited an increase in lipid drop formation of approximately 62% compared to that in Vxy-puro cells, suggesting that both *Pdk1* and *Pdk2* stimulate adipogenesis (Fig. 4a, b). In addition, *Pdk1* or *Pdk2* overexpression increased both the mRNA and protein expression levels of adipogenic marker genes, including C/EBPα, PPARγ, and SREBP-1c (Fig. 4c, Supplementary Fig. 5c,d). Individual knockdown of *Pdk1* or *Pdk2* resulted in a modest (10~ 20%) loss of adipogenic capacity, as evidenced by decreased lipid accumulation (Supplementary Fig. 5a, b), suggesting that PDK1 and PDK2 may act synergistically during adipogenesis. Indeed, profound inhibition of adipogenesis was observed when *Pdk1* and *Pdk2* were simultaneously knocked down in 3T3-L1 cells (Fig. 4d, e). Major adipogenic genes were also dramatically downregulated (Fig. 4f, Supplementary Fig. 5e, f) by *Pdk1* and *Pdk2* deficiency.

To further confirm that PDK plays important roles during adipogenesis, 3T3-L1 cells were treated with the pan-PDK inhibitor dichloroacetate (DCA) during adipocyte differentiation. DCA inhibited the phosphorylation of PDHE1α and the differentiation of 3T3-L1 cells in a dose-dependent manner (Fig. 4g, h and Supplementary Fig. 5g).

### Lactate-mediated stabilization of HIF1α is a key regulator of PDK-induced adipogenesis

Glucose, an essential substrate for triacylglycerol (TAG) storage in WAT, is used for de novo fatty acid synthesis and is converted to lactate during adipogenesis^18^. Lactate production from glucose is dramatically increased in adipose tissue in obese and diabetic humans^19^ and is known to be closely related to obesity and insulin resistance^20^.

To determine whether glycolysis is correlated with adipogenesis, we performed gene set enrichment analysis (GSEA) on the gene expression profile of differentiated 3T3-L1 cells using public databases^11^. The GSEA results showed a strong positive correlation between the expression of genes related to the glycolysis pathway (Supplementary Fig. 6a), such as *Hk2, Pgk1, Gapdh, Pkm*, and *Ldha* (Supplementary Fig. 7), and adipogenesis. Consistent with these findings, the extracellular acidification rate (ECAR) was increased in differentiated 3T3-L1 cells compared with undifferentiated cells (day 0) (Supplementary Fig. 6b). Lactate production was increased in differentiated 3T3-L1 cells (Supplementary Fig. 6c).

**Fig. 5:**
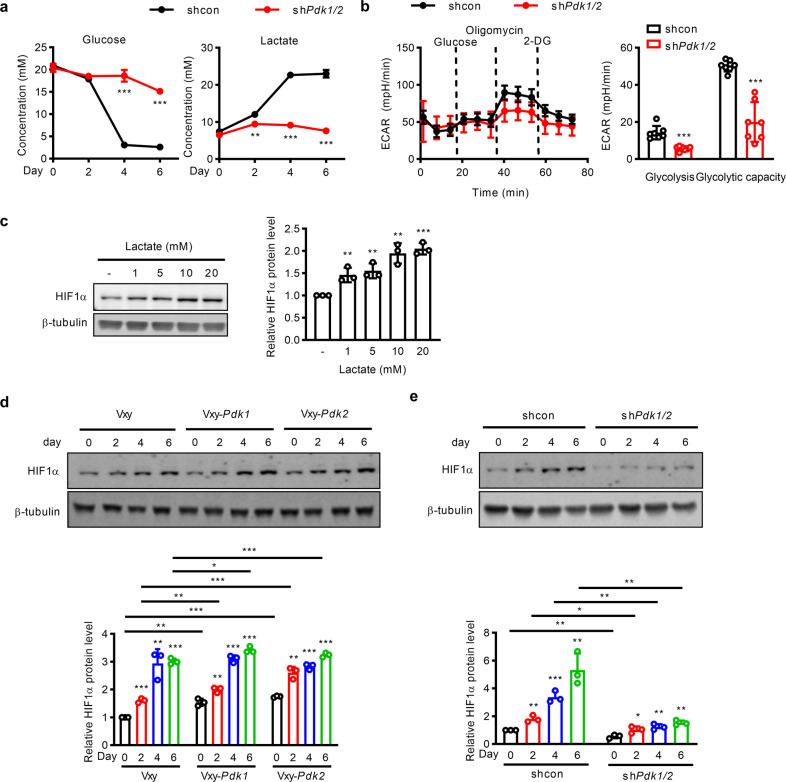
PDKs enhance HIF1α stability, leading to aerobic glycolysis during adipogenesis. **a** Glucose and lactate concentrations were measured in culture supernatant from control and *Pdk1/2*-silenced 3T3-L1 cells (*n* = 3). **b** Extracellular acidification rates (ECARs) measured in control and *Pdk1/2*-silenced 3T3-L1 cells (*n* = 7). **c** 3T3-L1 cells were exposed to increasing concentrations of lactate over a 24-hr period. The protein expression of HIF1a was measured in lactate-treated 3T3-L1 cells. **d**, **e** HIF1a protein expression was measured in 3T3-L1 cells with **d** stable *Pdk1* or *Pdk2* overexpression or **e** *Pdk1/2* silencing during adipocyte differentiation. The data are presented as the mean ± SD values. *P < 0.05, **P < 0.01, ***P < 0.001 by 2-tailed Student’s *t* test.

Because lactate plays an important role in adipogenesis, we tested whether glucose consumption and lactate production are regulated by PDK1/2 during adipogenesis. Concomitant increases in lactate production and glucose consumption were observed in 3T3-L1 cells approximately 4 days after the initiation of differentiation (Fig. 5a), consistent with previous data indicating that most lactate is formed from glucose^21^. The rapid glucose consumption and lactate production observed between 2–4 days after initiation of adipocyte differentiation corresponded chronologically to the timepoint at which PDK1 and PDK2 were induced (Fig. 3a–c), suggesting that enhanced glycolysis during adipogenesis may be related to the induction of PDKs (Fig. 5a). As expected, the median lactate levels and glucose levels did not change significantly in sh*Pdk1/2* 3T3-L1 cells (Fig. 5a). Similarly, the ECAR was significantly reduced in *shPdk1/2* 3T3-L1 cells (Fig. 5b). These results suggest that PDK1 and PDK2 are entirely responsible for glucose consumption and lactate production during adipocyte differentiation, prompting us to examine the effect of lactate on adipogenesis.

Lactate supplementation increased lipid droplet formation in a dose-dependent manner (Supplementary Fig. 6e). One of the well-established roles of lactate in metabolic reprogramming is accomplished by HIF1α activation^22^. Given that HIF1α can also mediate aerobic glycolysis^23^, we tested whether lactate can stabilize HIF1α in 3T3-L1 cells. Lactate dramatically increased the HIF1α protein level in a dose-dependent manner (Fig. 5c) but did not increase the mRNA level (data not shown), suggesting a role of lactate in HIF1α protein stabilization. The lactate level in the medium was increased by overexpression of either PDK1 or PDK2 (Supplementary Fig. 6d). In addition, the protein level of HIF1α increased continuously during cell differentiation and increased further in the presence of PDK1 or PDK2 (Fig. 5d).

**Fig. 6:**
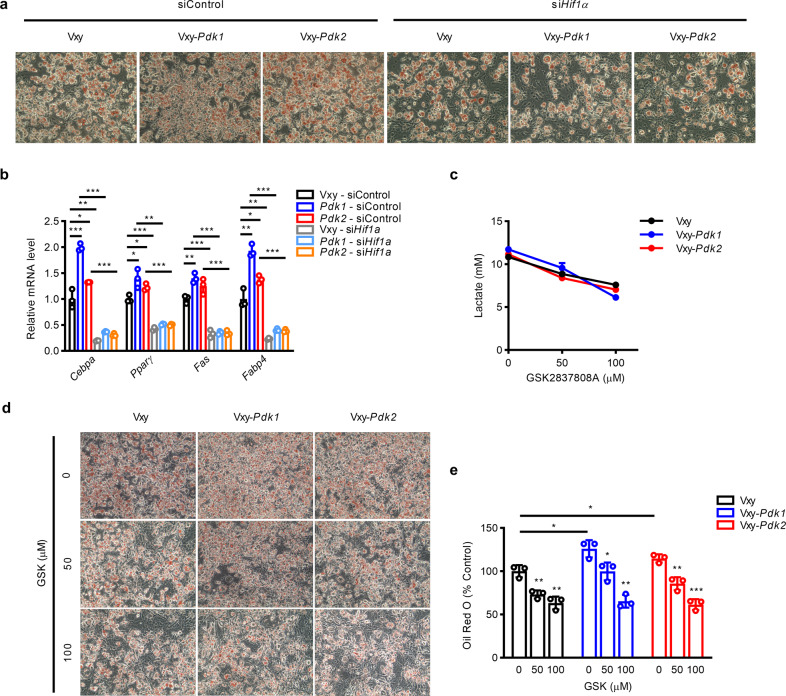
Inhibition of HIF1α expression or lactate production decreases adipogenesis in 3T3-L1 cells with stable *Pdk1* or *Pdk2* overexpression. **a** Oil Red O staining showed the effect of si*Hif1a* on adipogenesis in 3T3-L1 cells stably overexpressing *Pdk1* or *Pdk2*. **b** The mRNA expression levels of adipogenic genes were measured on day 6 after induction of differentiation (*n* = 3). **c** Extracellular lactate level in 3T3-L1 cells stably overexpressing *Pdk1* or *Pdk2* and treated with the LDH inhibitor GSK2837808A. **d**, **e** Lipid accumulation was evaluated by Oil Red O staining in 3T3-L1 cells stably overexpressing *Pdk1* or *Pdk2* with or without GSK2837808A treatment. The data are presented as the mean ± SD values. *P < 0.05, **P < 0.01, ***P < 0.001 by 2-tailed Student’s *t* test.

Consistent with these findings, HIF1α stability was not increased during adipogenesis in sh*Pdk1/2* cells (Fig. 5e), indicating that PDK1 and PDK2 are indispensable for HIF1α protein stabilization. Inhibition of HIF1α by chetomin or siRNA decreased adipogenesis mediated by PDK1 or PDK2 (Fig. 6a, Supplementary Fig. 6f), and adipogenic gene expression was suppressed by HIF1α knockdown (Fig. 6b).

Finally, to confirm that PDK1/2-mediated lactate production is responsible for adipogenesis, the LDH inhibitor GSK2837808A was used. LDH inhibitor treatment successfully decreased the lactate level in the medium (Fig. 6c), leading to a reduction in adipogenesis (Fig. 5d,e); these findings suggest that the PDK-lactate-HIF1α axis plays an important role in adipogenesis.

### The expression of PDK1 and PDK2 correlates positively with that of *PPARγ* in human adipose tissue in a manner dependent on the body mass index

As both global and adipose tissue-specific *Pdk2*^-/-^ mice are leaner than WT mice and *Pdk1/2* expression is increased during adipogenesis due to epigenetic modifications (Fig. 3), we analyzed the expression of PDK1/2 in human adipose tissue.

**Fig. 7:**
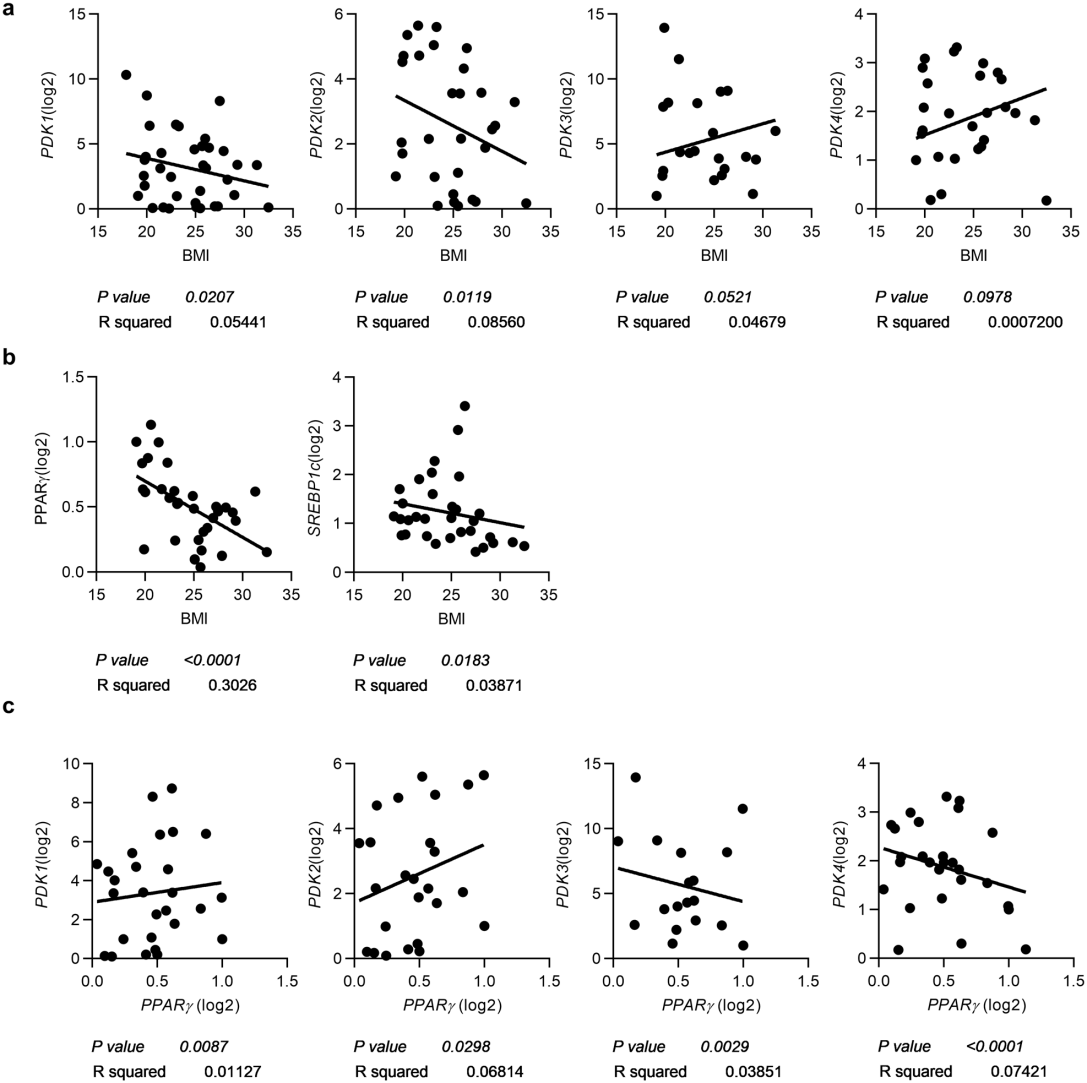
PDK1 and PDK2 expression levels are decreased in human visceral adipose tissue and are positively correlated with the PPARγ expression level. **a** Correlations between the mRNA expression of *PDK1, PDK2, PDK3*, and *PDK4* and body mass index (BMI). **b** Correlations of *PPARγ* and *SREBP-1c* mRNA expression with BMI. **c** Correlations between the expression of *PDK* isoforms and that of *PPARγ*.

*PPARγ* expression leads to the development of an adipogenic phenotype and is required for the differentiation of adipocytes in vivo. However, previous results showed that in mice, HFD feeding increases the levels of adipogenic genes such as *PPARγ*^24^ and lipogenic genes such as *SREBP-1c*^25^, which are paradoxically decreased in obese humans. To determine whether this pattern is also seen with *PDKs*, we analyzed the mRNA expression of various genes, including *PPARγ*, *SREBP-1c*, and *PDK* isotypes, in visceral adipose tissue from 34 subjects who had undergone nephrectomy as donors for kidney transplantation. qPCR analysis (bivariate analysis) revealed that the gene expression levels of *PDK1* and *PDK2* but not *PDK3* and *PDK4* correlated inversely with body mass index (BMI) in this cohort (Fig. 7a) (clinical and laboratory data are summarized in Supplementary Table 2).

In agreement with a previous series of studies^24^, the *PPARγ* gene expression level showed an inverse correlation with BMI. Furthermore, the levels of lipogenic genes such as *SREBP-1c* were inversely correlated with BMI (Fig. 7b). In this cohort, a positive correlation between the *PDK1/2* and *PPARγ* expression levels was noted (Fig. 7c), suggesting that like *PPARγ* expression, *PDK1* and *PDK2* expression might be implicated in the pathogenesis of human obesity. In addition, comparison of the protein levels of PDK1 and PDK2 in adipose tissue from normal chow-fed and high-fat diet-fed mice revealed that the levels of these proteins were decreased in mice fed a HFD (Supplementary Fig. 8).

## Discussion

In the present study, we observed that body weight gain and adipose tissue mass in *Pdk*2^-/-^ mice were significantly lower than those in WT mice upon HFD feeding. In addition, adipose tissue-specific *Pdk*2 deficiency was sufficient to decrease adipose tissue mass independent of changes in whole-body energy expenditure. In 3T3-L1 cells, the mRNA expression levels of *Pdk1* and *Pdk2* were increased by epigenetic regulation, and the corresponding protein expression levels were increased due to an increase in lactate production during adipocyte differentiation. Stable overexpression of *Pdk1* or *Pdk2* in 3T3-L1 cells stimulated adipocyte differentiation, whereas knockdown of *Pdk1/2* or pharmacological inhibition of PDK activity with DCA attenuated adipocyte differentiation. These results suggest that PDK1 and PDK2 play a critical role in fat accumulation.

Previous studies have shown that HIF1α protein expression is increased in differentiated 3T3-L1 cells as well as in adipose tissue of subjects with obesity^26^. As shown in Fig. 5, HIF-1α protein expression was elevated in *Pdk1-* or *Pdk2*-overexpressing 3T3-L1 cells and decreased in sh*Pdk1/2* cells during adipocyte differentiation. Metabolic conversion of glucose to lactate was increased during adipocyte differentiation in 3T3-L1 cells but was hampered by PDK1/2 deficiency. This effect was dependent on the inhibition of lactate production and subsequent inhibition of HIF1α stabilization. Lactate production is increased during adipocyte differentiation^21^. In the present study, exogenous lactate supplementation induced adipocyte differentiation. Although the mechanism by which lactate promotes adipocyte differentiation remains largely unknown, our study revealed that HIF1α stabilization by lactate may be one of the key mechanisms linking lactate accumulation and adipocyte differentiation via PDK1/2. This possibility is supported by our finding that genetic and pharmacologic inhibition of HIF1α abrogated adipocyte differentiation in either the presence or absence of PDK1/2 overexpression (Fig. 5e, Supplementary Fig. 6f). Furthermore, the LDH inhibitor phenocopied the effect of the HIF1α inhibitor on adipogenesis (Fig. 6d,e). Accumulating evidence shows that increased lactate production, a result of metabolic reprogramming by PDK induction, can mediate HIF-1a stabilization^27,28^. Furthermore, in a previous paper^29^, it was proposed that overexpression of C/EBPδ and HIF-1A upregulates the promoter activity of adipocyte-specific genes such as leptin, CFD, HIG2, LPL, and PGAR.

In conjunction with these findings, it should also be noted that HIF1α is the primary regulator of glycolysis (Supplementary Fig. 7). As both PDK1 and PDK2 are known to be transcriptionally regulated by HIF1α^30^, not only the PDK1/2-lactate-HIF1α axis but also the inverse axis might play a metabolic signaling role in adipogenesis.

We sought to identify the putative transcription factors that can regulate the transcription of PDK1 and PDK2 during adipocyte differentiation by iRegulon analysis based on microarray and ChIP-seq data (Supplementary Tables 3 and 4)^31^. Interestingly, we found that JUN and FOS might regulate the transcription of PDK1 and PDK2 during adipocyte differentiation. Previous research has suggested that the expression of JUN and FOS is increased together with that of Myc and C/EBP within 1 h after the addition of methylisobutylxanthine, dexamethasone, and insulin^32,33^ and that transgenic mice expressing a dominant-negative protein that prevents binding to the basic leucine zipper domain of JUN family proteins under the control of the adipose-enriched aP2 enhancer/promoter lack white adipose tissue^34^. It has also been reported that PPARγ expression is synergistically upregulated by JUN and FOS^35^. Considering these observations collectively, we hypothesized that JUN and FOS might activate the transcription of PDK1 and PDK2 in the initial phase of adipocyte differentiation. Increased expression of PDK1 and PDK2 by JUN and FOS enforces HIF1a stability to facilitate adipocyte differentiation and obesity.

One of the unexpected findings in the current study was the involvement of PDK1 in adipogenesis. Unlike the roles of PDK2 and PDK4, the role of PDK1 is underexplored in obesity and diabetes. Unlike in vivo studies that demonstrated that adipose tissue-specific PDK2 deficiency was sufficient to slow adiposity upon HFD feeding, our in vitro studies revealed that deletion of PDK1 or PDK2 alone in 3T3-L1 cells was not sufficient to prevent adipogenesis, suggesting that substantial induction of PDH by dual inhibition of PDK1 and PDK2 might be required for sufficient metabolic reprogramming of pyruvate flux into acetyl-CoA to reduce lactate production. Further evaluation of adipose tissue mass in adipose tissue-specific *Pdk1*^*-/-*^ rodents would enhance our understanding of the importance of PDK1 in adipogenesis under pathological conditions. Further inhibition of adipogenesis is anticipated in adipose tissue-specific *Pdk1/2*^*-/-*^ mice, which were not available for the current study.

We observed a positive correlation between PPARγ and PDK1/2 expression in visceral adipose tissue obtained from individuals with various degrees of obesity. Similarly, PDK1/2 protein levels were decreased in mice fed a HFD compared with mice fed a normal chow diet. Recent literature has shown that chronic HFD feeding in rodents impairs the expression of adipogenic differentiation-related genes^36^. Similar findings have also been reproduced in humans^37^. Notably, whereas PDK1/2 expression was decreased, PDK4 expression was robustly increased in adipose tissue of both HFD-fed mice and obese humans (Fig. 6 and Supplementary Fig. 8). Under fasting or HFD-fed conditions, when glucose uptake by adipose tissue is disrupted by insulin deficiency or insulin resistance, respectively, glyconeogenesis in adipose tissue relies on pyruvate derived from lactate or alanine. This ability requires inhibition of PDC activity by induction of PDK4^38^. In obesity, adipogenic differentiation is typically insufficient to meet metabolic demands, and excess calories are primarily stored in preexisting adipocytes, which become overloaded with lipids. This process could explain the decrease in PDK1/2 expression and increase in PDK4 expression in the adipose tissue of individuals with severe obesity.

In summary, PDK2 deficiency in adipose tissue protects against adiposity induced by HFD feeding without reducing glucose tolerance and simultaneously improves insulin sensitivity. In vitro studies demonstrated that PDK1 and PDK2 regulate adipocyte differentiation by enhancing HIF1α expression through lactate-mediated protein stabilization. As the expression of *PDK*s has been noted in the adipose tissue of humans, pharmacological inhibition of PDK1/2 might also play a critical role in the prevention and treatment of obesity in humans.

## Supplementary information

Supplementary Information
