# Supplementary material for: Pyruvate dehydrogenase kinase 1 and 2 deficiency reduces high-fat diet-induced hypertrophic obesity and inhibits the differentiation of preadipocytes into mature adipocytes
Source: Exp Mol Med. 2021 Sep 22;53(9):1390–401. doi: 10.1038/s12276-021-00672-1 (PMC8492875; doi:10.1038/s12276-021-00672-1)
Supplement: Supplementary file 1 — Supplementary Information [file 12276_2021_672_MOESM1_ESM.docx]

**Supplementary Data**

**Supplementary Material and Methods**

**Measurement of energy expenditure using an indirect calorimetry system**

CO2 production rates, O2 consumption rates, energy expenditure (EE), and physical activity were measured with a TSE indirect calorimetry system (LabMaster, TSE systems, Bad Homburg, Germany) for 24 h. Mice were individually housed in metabolic cages with 12 h light/dark cycles at 22 ± 2°C.

**Primary adipocytes isolation and differentiation**

Stromal vascular fraction (SVF) was isolated from subcutaneous fat. Subcutaneous fat from 3-to 4- week old mice were excised and minced in collagenase buffer. Tissue was digested in 37°C with constant agitation at 160 rpm for 30min. Cell suspension was filtered through cell strainer (100 µm) and centrifuged at 1500 rpm for 5min. Re-suspended SVF cells were filtered through 40 µm cell strainer and centrifuged at 1500 rpm for 5min. SVF cells were plated in high glucose-DMEM supplemented with 10% bovine serum. Preadipocytes were induced to differentiate using medium supplemented with 10% FBS, 0.5 mM IBMX, 1 uM dexamethasone, 5 ug/ml insulin and 1 uM rosiglitazone. After 2days, the medium was replaced with medium containing 10% FBS supplemented with 1 ug/ml insulin, and changed every 2days thereafter with medium containing 10% FBS.

**Measurement of extracellular acidification rate (EACR)**

The extracellular acidification rate (ECAR) were measured using an XFe96 Extracellular Flux Analyzer (Seahorse Bioscience, Billerica, MA, USA) according to the manufacturer’s protocol. 3T3-L1 cells were seeded in 0.2% gelatin coated XFe96 cell culture plates at a density of 3 x 10^3^ cells/well. Two day after plating, the growth medium was replaced with differentiation medium for adipogenic differentiation. After 6days, the medium was changed to XF base medium (pH 7.4, Seahorse Biosciences) with 1 mM glutamine (Sigma-Aldrich; G8540). To assess ECAR, the compounds and metabolites used in this study were as follow: Glucose (10 mM, Sigma-Aldrich; G7528), oligomycin A (2 μM, Sigma-Aldrich; 75351), and 2-DG (2-Deoxy-D-glucose, 50 mM, Sigma-Aldrich; D6134). For the normalization, DAPI stained cells were counted by the microscopy automatically (BioTek Lionheart FX, The Netherlands).

**Supplemental Figures and Legends**

**
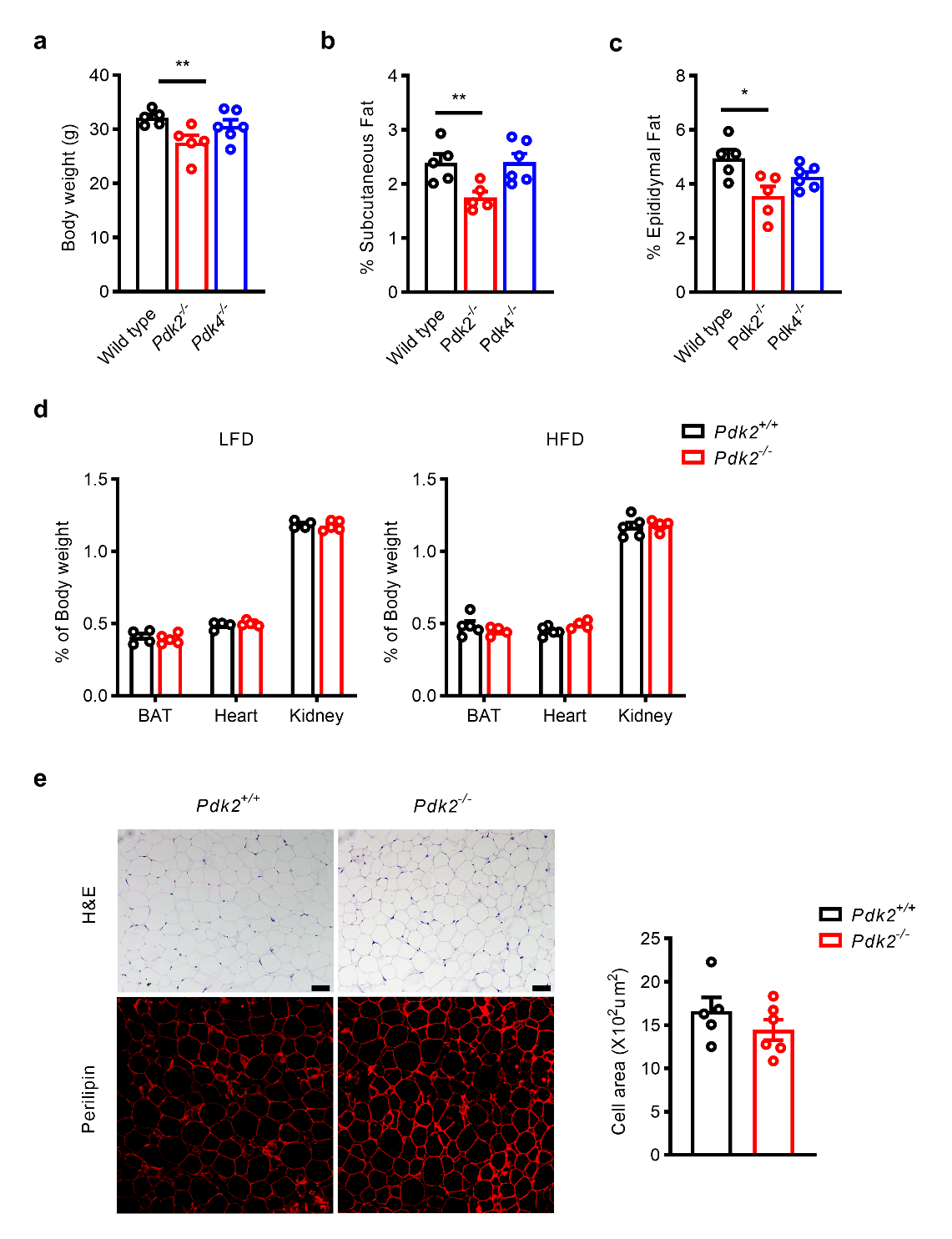
**

**Supplementary Figure 1. *Pdk2* knockout mice gained less body weight and fat mass on HFD.** (a) Body weight, (b) subcutaneous and (c) epididymal adipose tissue weight (% of body weight) were determined from WT, *Pdk2^-/-^* or *Pdk4^-/-^* mice fed HFD for 6 weeks (n=5-6 per group). (d) Tissue weight (% of body weight) were determined from WT and *Pdk2^-/-^* mice fed LFD or HFD for 4 weeks (n=4-5 per group). (e) H&E and perilipin staining of epididymal adipose tissue from WT and *Pdk2^-/-^* mice fed LFD for 4 weeks (n = 5-6 per group). Adipocyte areas were determined with Image J. Data are presented as mean ± SEM. **P* < 0.05; ***P* < 0.01, 1-way ANOVA followed by LSD test.


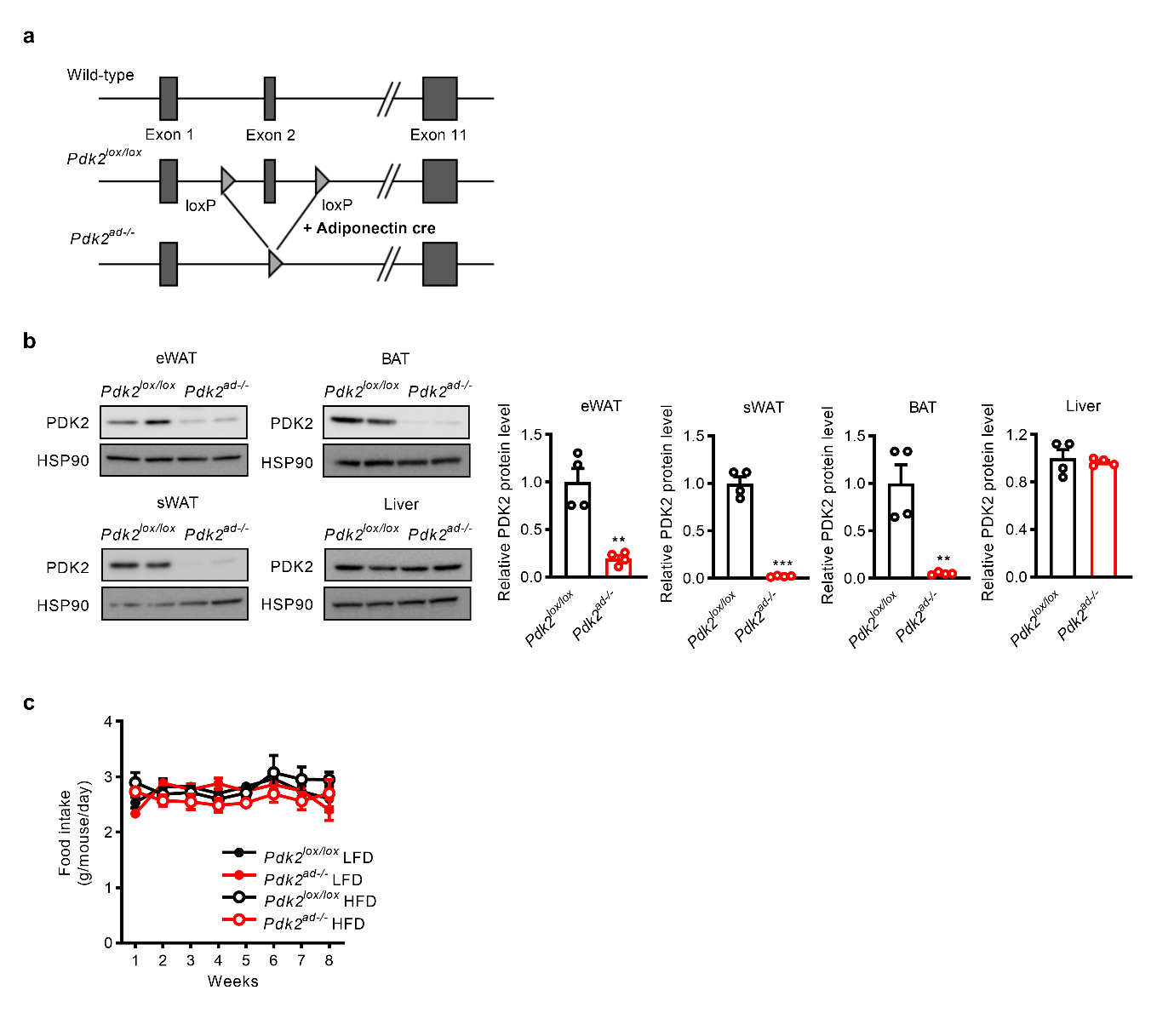


**Supplementary Figure 2. Generation of Adipose tissue specific *Pdk2* knockout mice and their food intake** (a) Scheme of targeting strategy. Adipocyte-specific gene knockout strategy, with triangles designating LoxP sites flanking the exon 2 of PDK2. (b) PDK2 protein expression in tissues of *Pdk2^lox/lox^* and *Pdk2^ad-/-^* mice fed a standard chow diet was measured by western blotting. (c) Food intake was measured in *Pdk2^lox/lox^* and *Pdk2^ad-/-^* mice fed a LFD or HFD for 8 weeks. Data are presented as mean ± SEM. ***P* < 0.01; ****P* < 0.001, 2-tailed Student’s *t* test.


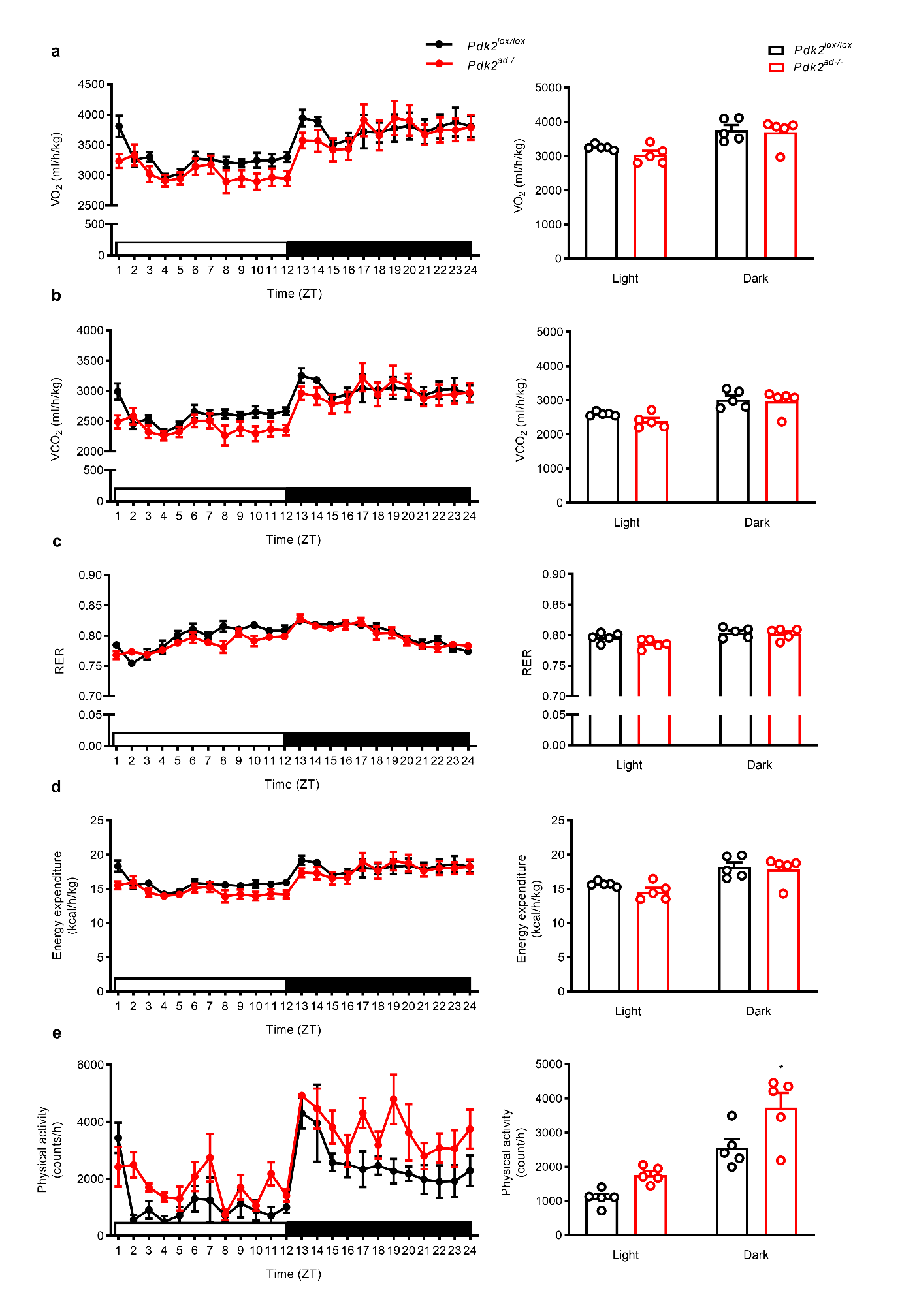


**Supplementary Figure 3. Adipose tissue specific *Pdk2* knockout mice show comparable energy expenditure compared with WT.** The metabolic parameters were measured using metabolic phenocages in *Pdk2^lox/lox^* and *Pdk2^ad-/-^* fed HFD for 5 weeks. (a) VO_2_, (b) VCO_2_, (c) respiratory exchange ratio (RER), (d) energy expenditure and (e) physical activity were measured and it was represented by light cycle and dark cycle as separately (n=5 per each group). Data are presented as mean ± SEM. **P* < 0.05, 2-tailed Student’s *t* test.

**
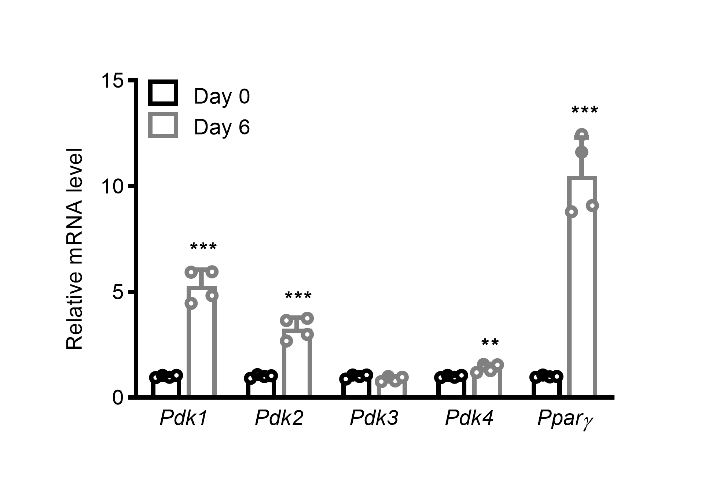
**

**Supplementary Figure 4. Expression of PDKs in primary adipocyte.** Primary stromal vascular fractions (SVFs) isolated from mouse subcutaneous fat were differentiated into mature adipocytes. mRNA expression of *Pdk* isoforms and *Pparγ* were measured at day 0 and day 6 after induction of differentiation (n=4). Data are presented as mean ± SD. ***P* < 0.01; ****P* < 0.001, 2-tailed Student’s *t* test.

**
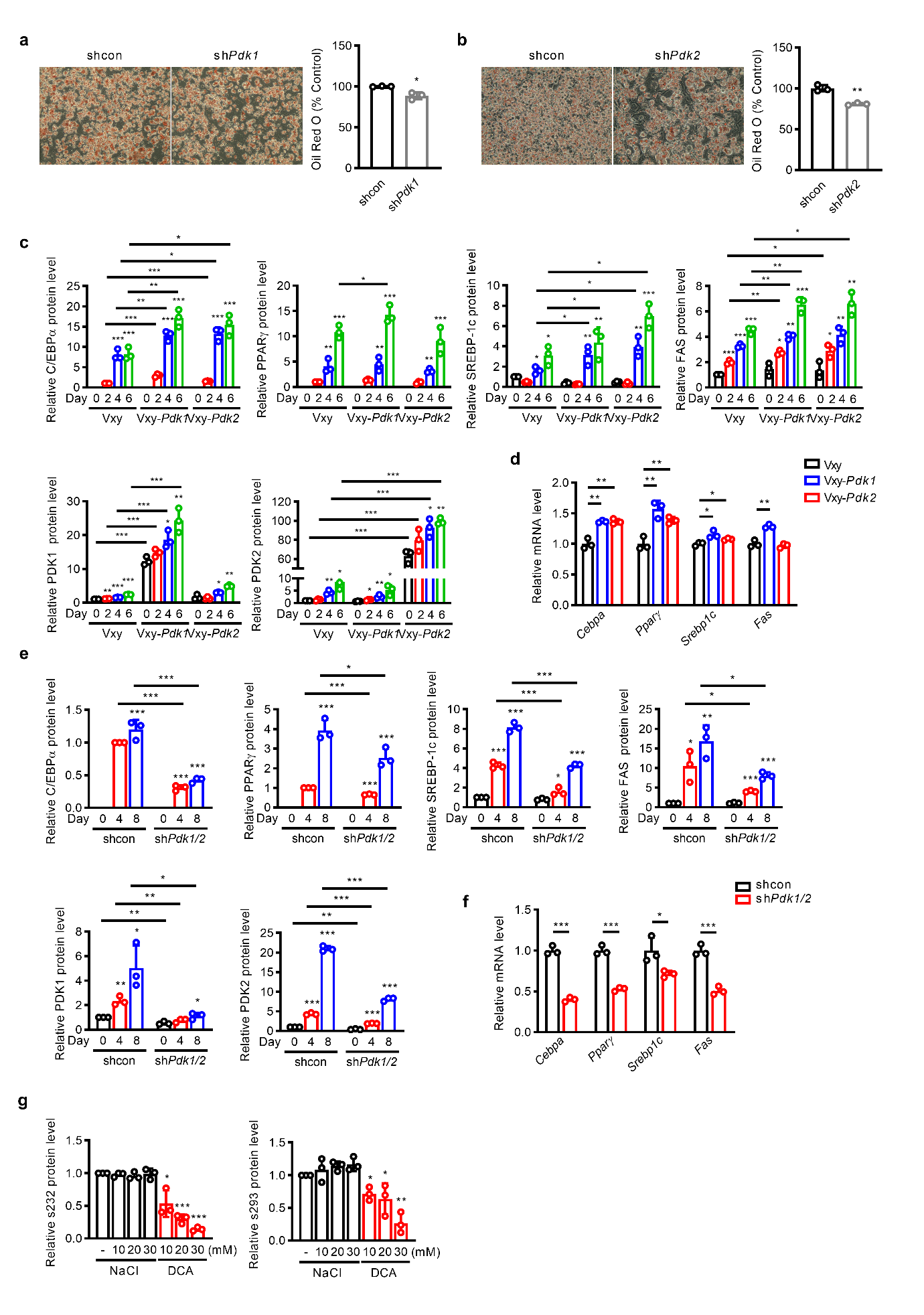
 Supplementary Figure 5. Deficiency of PDK1 or PDK2 decrease adipogenesis in 3T3-L1 preadipocytes.** (a) *Pdk1* or (b) *Pdk2* knockdown 3T3-L1 cells were differentiated in the differentiation medium for 6 days. Intracellular lipid accumulation was determined by Oil- Red-O staining (n=3). (c) The relative protein and (d) mRNA expression levels of adipocyte-specific genes were measured in 3T3-L1 cells stably expressing Vxy-puro, Vxy- *Pdk1*, or Vxy- *Pdk2*. (e) The relative protein and (f) mRNA expression levels of adipocyte-specific genes were measured in control and *Pdk1/2*-silenced 3T3-L1 cells. (g) The phosphorylation levels of PDHE1a were measured in differentiated 3T3-L1 cells. Data are presented as mean ± SD. **P* < 0.05; ***P* < 0.01; ****P* < 0.001, 2-tailed Student’s *t* test.

**
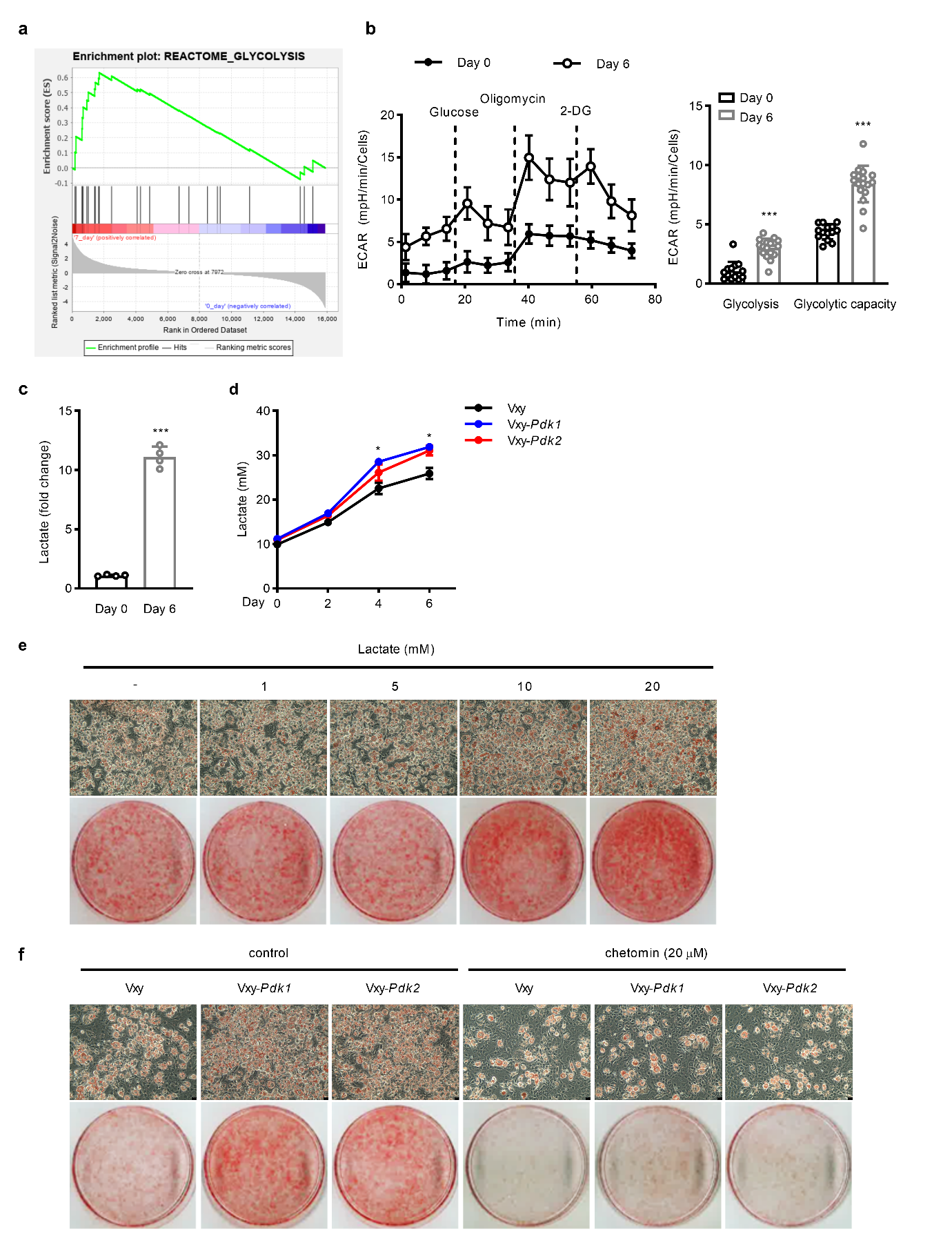
**

**Supplementary Figure 6. Glycolysis is increased during adipogenesis.** (a) Enrichment of glycolysis pathway confirmed by Gene Set Enrichment Analysis (GSEA) during differentiation in 3T3-L1 cells using public microarray data set (GSE 20752). (b) Extracellular acidification rates (ECARs) was measured in undifferentiated or differentiated 3T3-L1 cells. (c) Lactate levels were measured in cell culture media of undifferentiated or differentiated 3T3-L1 cells (n = 4). (d) Lactate levels were measured in cell culture media of 3T3-L1 cells stably expressing Vxy-puro, Vxy- *Pdk1*, or Vxy- *Pdk2* in day 0, 2, 4, and 6 after differentiation (n=3). (e) Two-day post-confluent 3T3-L1 preadipocytes were cultured with differentiation medium in the absence or presence of lactate at the indicated dose for 6 days. On day 6, intracellular lipid accumulation was determined by Oil-Red-O staining. (f) Oil-Red-O staining showed the effect of chetomin on adipogenesis in *Pdk1* or *Pdk2*-stably overexpressed 3T3-L1 cells. Data are presented as mean ± SD. **P* < 0.05; ****P* < 0.001, 2-tailed Student’s *t* test.


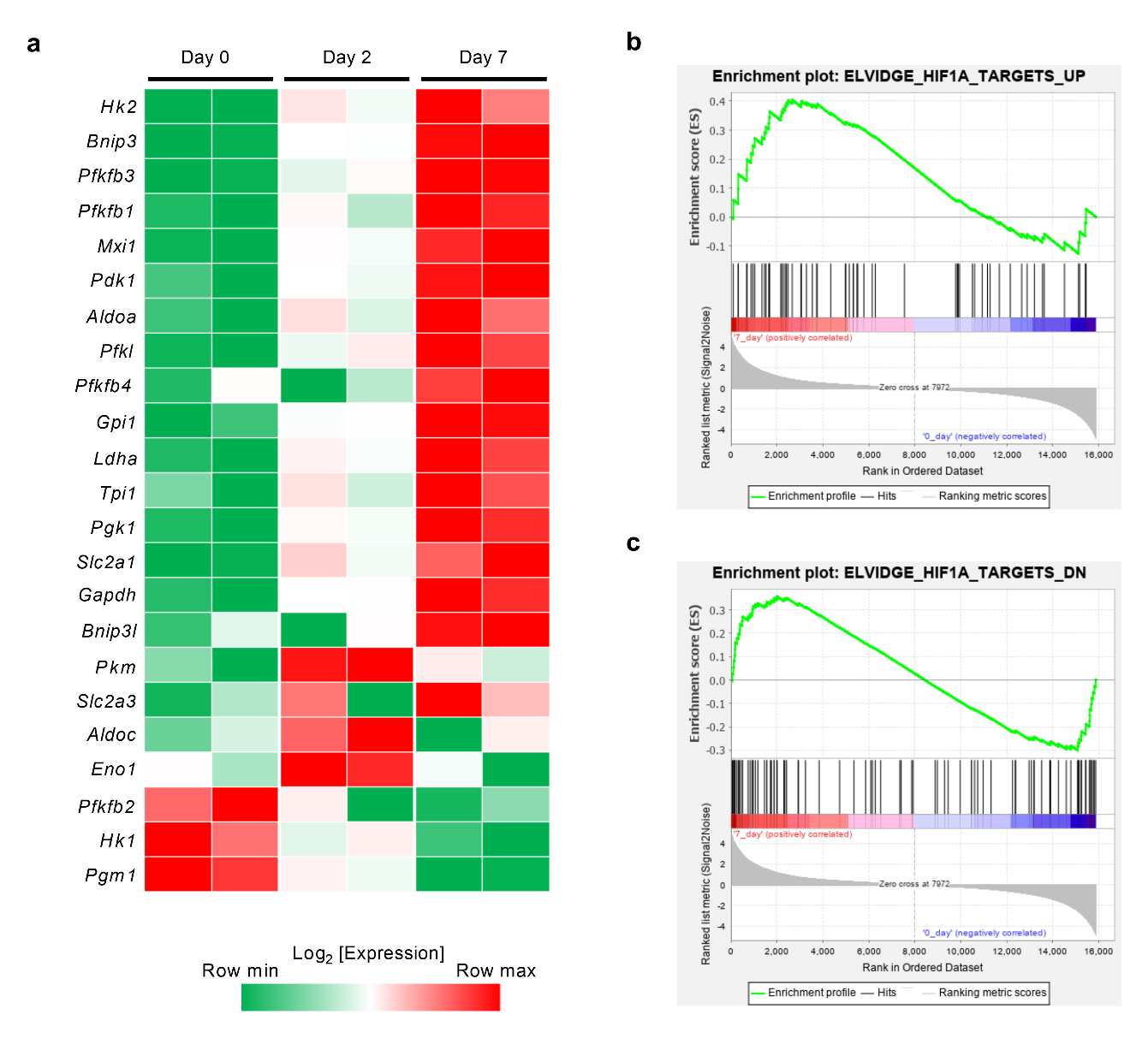


**Supplementary Figure 7. HIF1a-mediated glycolysis is increased during adipocyte differentiation.** (a) Glycolysis related HIF1a target gene expression levels during 3T3-L1 cell differentiation were analyzed from public microarray data (GSE 20752). (b) Positively correlated or (c) negatively correlated genes by HIF1a were analyzed using GSEA.


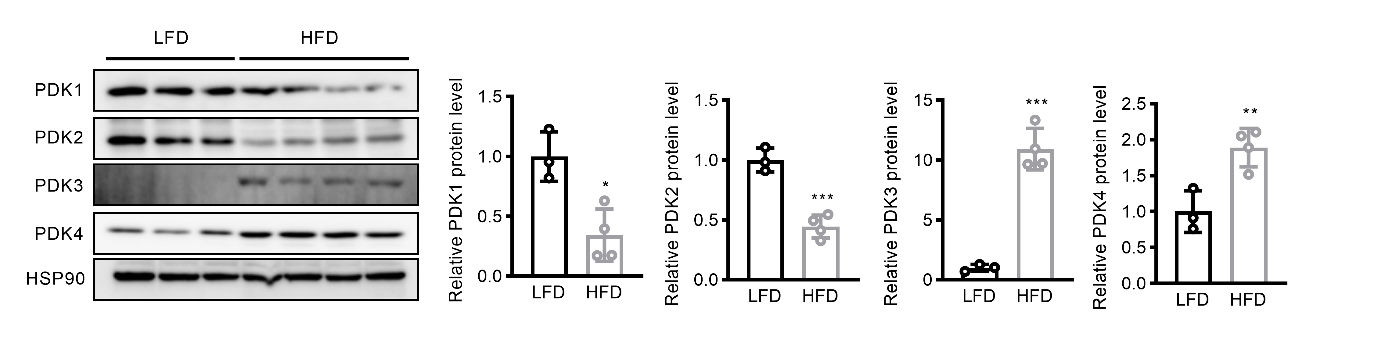


**Supplementary Figure 8. PDK1 and PDK2 expressions are decreased in long term HFD induced obese mice**. Protein expression levels of PDK isoforms were observed in epididymal fat tissue of WT mice fed LFD or HFD for 16 weeks.

**Supplementary Table 1. Primer sequence for real-time PCR**

| Gene | Accession No. | Sequence |
| --- | --- | --- |
| Mouse |  |  |
| *Pdk1* | NM_172665 | Forward CACCACGCGGACAAAGG  Reverse GCCCAGCGTGACGTGAA |
| *Pdk2* | NM_133667 | Forward CCCCGTCCCCGTTGTC  Reverse TCGCAGGCATTGCTGGAT |
| *Pdk3* | NM_145630 | Forward GGAGCAATCCCAGCAGTGAA  Reverse TGATCTTGTCCTGTTTAGCCTTGT |
| *Pdk4* | NM_013743 | Forward CCATGAGAAGAGCCCAGAAGA  Reverse GAACTTTGACCAGCGTGTCTACAA |
| *Cebpa* | NM_007678.3 | Forward GCGCAAGAGCCGAGATAAAG  Reverse CGGTCATTGTCACTGGTCAACT |
| *Pparγ* | NM_011146.3 | Forward CACAAGAGCTGACCCAATGGT  Reverse GATCGCACTTTGGTATTCTTGGA |
| Srebp1c | NM_011480 | Forward GGCCGAGATGTGCGAACT  Reverse CCCGGGAAGTCACTGTCTTG |
| *Fas* | NM_007988.3 | Forward ACCTGGTAGACCACTGCATTGAC  Reverse CCTGATGAAACGACACATTCTCA |
| *Fabp4* | NM_024406.3 | Forward CCATCCGGTCAGAGAGTACT  Reverse CGAATTCCACGCCCAGTT |
| *36B4* | NM_007475 | Forward ACCTCCTTCTTCCAGGCTTT  Reverse CTCCAGTCTTTATCAGCTGC |
| Human |  |  |
| *PDK1* | NM_001278549.2 | Forward GGCCAGGTGGACTTCTACG  Reverse TGAACGGATGGTGTCCTGAG |
| *PDK2* | NM_002611.5 | Forward TACGTCCCCTCCCACCTCTA  Reverse GTCGCCCTCATGGCATTCT |
| *PDK3* | NM_001142386.3 | Forward CCAGCCTGGAGCCTACCA  Reverse CGGGAAATTGGCAAACCATA |
| *PDK4* | NM_002612.4 | Forward GTATGTTCCTTCTCACCTCC  Reverse TGTTGCCCGCATTGCAT |
| *PPARγ* | NM_138712.3 | Forward AGCCTCATGAAGAGCCTTCC  Reverse TCCGGAAGAAACCCTTGCA |
| *SREBP-1c* | NM_001321096.3 | Forward GGTGCAGACAGGACAGTGTT  Reverse CCCTACTCCCAGACTGCTCT |
| *Cyclophilin* | NM_021130.5 | Forward TCTGCACTGCCAAGACTGAG  Reverse TCGAGTTGTCCACAGTCAGC |

**Supplementary Table 2. Baseline characteristics of the enrolled patients**

|  | **Male** | **Female** |
| --- | --- | --- |
| **Enrollment period** | Aug. 2015 ~ June 2018 | |
| **Number** | 15 | 19 |
| **Age** | 41.6 ± 16.33 | 46.6 ± 8.25 |
| **Height, cm** | 170.7 ± 5.90 | 160.1 ± 5.39 |
| **Body weight, kg** | 74.6 ± 13.61 | 60.8 ± 8.45 |
| **BMI** | 25.4 ± 3.55 | 23.8 ± 3.41 |
| **Blood glucose, mg/dL** | 120.1 ± 23.19 | 112.5 ± 22.80 |

Supplementary Table 3. List of the putative transcription factor in PDK1 promoter during adipocyte differentiation

| **Rank** | **AUC** | **NES** | **ClusterCode** | **Transcription factor** | **Target genes** |
| --- | --- | --- | --- | --- | --- |
| 1 | 0.223468 | 4.76465 | T11 | JUN | PDK1 |
| 2 | 0.217489 | 4.62899 | T11 | JUN | PDK1 |
| 3 | 0.212257 | 4.5103 | T2 | FOS | PDK1 |
| 4 | 0.210015 | 4.45943 | T11 | JUN | PDK1 |
| 5 | 0.207773 | 4.40856 | T15 | BHLHE40 | PDK1 |
| 6 | 0.205157 | 4.34921 | T16 | TEAD4 | PDK1 |
| 7 | 0.204783 | 4.34073 | T11 | JUN | PDK1 |
| 8 | 0.200673 | 4.24747 | T8 | MAFK | PDK1 |
| 9 | 0.183483 | 3.85747 | T21 | GATA2 | PDK1 |
| 10 | 0.177877 | 3.7303 | T23 | FOSL1 | PDK1 |
| 11 | 0.171898 | 3.59464 | T24 | MAX | PDK1 |
| 12 | 0.169283 | 3.5353 | T25 | EP300 | PDK1 |
| 13 | 0.161809 | 3.36573 | T28 | GATA1 | PDK1 |

Supplementary Table 4. List of the putative transcription factor in PDK2 promoter during adipocyte differentiation

| **Rank** | **AUC** | **NES** | **ClusterCode** | **Transcription factor** | **Target genes** |
| --- | --- | --- | --- | --- | --- |
| 1 | 0.232436 | 4.96813 | T6 | NFYA | PDK2 |
| 2 | 0.228326 | 4.87487 | T8 | MAFK | PDK2 |
| 3 | 0.213752 | 4.54421 | T13 | GTF2F1 | PDK2 |
| 4 | 0.21151 | 4.49334 | T14 | IRF3 | PDK2 |
| 5 | 0.211136 | 4.48486 | T2 | FOS | PDK2 |
| 6 | 0.195067 | 4.1203 | T17 | MNT | PDK2 |
| 7 | 0.19432 | 4.10334 | T18 | FOXG1 | PDK2 |
| 8 | 0.185351 | 3.89986 | T20 | USF1 | PDK2 |
| 9 | 0.177877 | 3.7303 | T22 | SP1 | PDK2 |
